# The AERO prosthetic liner: socket pressure distribution, comfort and material composition

**DOI:** 10.1080/07853890.2024.2380798

**Published:** 2024-07-26

**Authors:** Yusuke Miyata, Kazuhiko Sasaki, Gary Guerra, Woratee Dacharux, Sirarat Chaisumritchoke

**Affiliations:** a Sirindhorn School of Prosthetics and Orthotics, Faculty of Medicine Siriraj Hospital, Mahidol University, Bangkok, Thailand; b Department of Exercise and Sport Science, St. Mary’s University, San Antonio, TX, USA; c Department of Anatomy, Faculty of Medicine Siriraj Hospital, Mahidol University, Bangkok, Thailand

**Keywords:** Prosthetic, liner, transtibial, pressure, socket comfort, resource limited, material testing

## Abstract

**Purpose:**

This study aimed to evaluate the pressure distribution and comfort of transtibial prosthesis wearers using an affordable ethyl-vinyl acetate (EVA) roll-on (AERO) liner.

**Method:**

Fifteen unilateral transtibial prosthesis users wore patella tendon bearing (PTB) sockets with a polyethylene foam (PE-lite) liner were enrolled this study. AERO liners were provided to all participants. Six force sensors were applied to the residual limb to evaluate pressure distribution during treadmill walking, and the socket comfort score (SCS) was used to evaluate comfortability. Fourier transform infrared (FT-IR) spectroscopy was performed on the EVA and PE-lite liners.

**Results:**

Eleven participants used prefabricated AERO liners and four participants used custom-made AERO liners. The pressure distribution was analysed by the coefficient of variation (CV): PE-lite was 75.7 ± 6.0 and AERO liner 83.3 ± 4.1. Residual limb pressure was significantly decreased when using the AERO liner (*p* = .0007), with a large effect size (*r* = 0.87). Mean SCS was 7.5 ± 1.3 and 8.9 ± 1.1 for PE-lite and AERO liner respectively.

**Conclusion:**

Better pressure distribution and comfort were observed when the participants used the AERO liner. AERO had a greater proportion of calcium carbonate (CaCO_3_). These findings suggest that the AERO liner is a better off-the-shelf option for persons using traditional prosthetic sockets and liners.

## Introduction

The number of transtibial amputations is increasing globally in low-income and middle-income countries [1]. For lower limb prosthesis users, mobility has been strongly associated with Quality of Life (QoL) [2]. Restriction of prosthesis use because of a lowered comfort and use, might alter participation. Roll-on prosthetic liners offer comfort but at a higher price point, excluding their potential use in resource limited settings [3,4]. Thus, in resource limited settings, prosthetic interventions that promote comfort and function may in fact be warranted [5]. The use of a prosthesis can potentially alleviate functional impairments following amputation, enabling individuals to walk and regain pre-amputation levels of activity. Various materials have been used in transtibial prostheses [6]. However, one of the most significant challenges in resource limited environments (RLE) is to gain access to prostheses that are both comfortable and inexpensive. The limitations of materials have long been a significant problem in RLE for transtibial prosthetic users for a long time [7–9]. An appropriate combination of prosthetic sockets and liner materials is important in transtibial prostheses. The patellar tendon bearing socket, also known as the PTB socket, is commonly used with polyethylene foam (PE-lite) liner material. The PTB design primarily accommodates patient weight-bearing in the proximal region of the residuum and the socket provides anterior and posterior compression. Some users prefer this liner and socket designs [10]. However, total surface bearing (TSB) prosthetic sockets are more effective in distributing pressure across the entire limb than PTB sockets. The TSB socket enhances prosthetic gait performance and facilitates ambulation [11–13]. Additionally, a gel liner, which is made of a softer material, is typically used to provide cushioning and accommodate the contours of residual limbs with the TSB socket [14,15]. Gel-based silicone and thermoplastic elastomer (TPE) liners with different hardnesses have been developed, and prosthetists select the liner based on the patient’s residual limb condition. For example, a TPE liner with greater cushioning and stretchability is utilized for bony residuums with less soft tissue or short residuum that lacks sufficient surface areas of the residual limb. Silicone helps provide coverage for soft tissue and protects the limb while improving the integrity of the prosthesis [16–19]. Therefore, these liners help to effectively protect and prevent skin breakdown and increase the suspension. In addition, thicker liners provide greater pressure distribution during walking [20]. However, even if a patient uses a gel liner, an ill-fitting prosthetic socket may add excessive pressure, causing skin [21].

Although these commercial liners have their merits and are available, there are individuals in low to middle-income countries that cannot afford them, and there may be barriers within the healthcare scheme for coverage of higher costing liners. One possible affordable option is ethyl-vinyl acetate (EVA), which is a closed-cell material similar to PE-lite. This material is widely used in a variety of products, such as insoles or as foam in an orthotic device and is affordable. An affordable ethyl-vinyl acetate roll-on (AERO) liner has been developed to improve function and user roll-on donning [22]. This liner uses locally sourced materials and is simple to make, making it a more affordable and sustainable prosthetic liner for RLE. Some preliminary pilot data of prosthesis users utilizing the AERO have shown that the liner improves prosthetic comfort, stability and pressure distribution for transtibial prostheses; however, there were limitations of a small sample size and recruitment of participants with ideal residual limb shapes [23]. The utility of AERO is that it can be custom-fabricated to the unique residuum of an existing prosthesis user without the need for fabrication of an entirely new prosthesis. However, this aspect has yet to be explored in a research study.

To understand the interaction between the residual limb and prosthesis, it is essential to evaluate the residual limb pressure. This may help to identify the potential risks of residual limb damage or skin breakdown. Moreover, to reduce the peak pressure on the residual limb, it is crucial to consider the correct socket design and liner material to mitigate peak pressures during walking, as this has a significant impact on the comfort of patients.

Therefore, this study aimed to examine the pressure distribution and socket comfort of transtibial prosthesis wearers while using either their standard-of-care PE-lite liner or AERO liner during walking. Moreover, the material properties of the AERO liner have not been determined. The second aim of this study was to perform material analysis using AERO to determine the chemical composition of the material.

## Method

### Participants

This study was approved by the Siriraj Faculty of Medicine Institutional Review Board (Si 419/2022). Fifteen individual transtibial prosthesis users were recruited for this study, and all participants signed a consent form indicating their agreement to participate. The inclusion criteria for this study included participants who did not have any underlying diseases that affected their daily activities. Good manual dexterity to require roll-on donning of AERO liner and currently uses a PTB transtibial prosthetic design with a rigid resin socket and a PE-lite liner material. An Amputee Mobility Predictor (AMPRO) score of at least K2 level was required because we require participants to walk for 2 min on a treadmill [24]. The participant’s own prosthetic foot, either a solid ankle cushion heel (SACH) or a single-axis foot, was used. Eight participants used the cuff strap suspension system, and seven used an anatomical supra-condylar suspension for their current device. The participants had a variety of residual limb conditions, such as short limb length, conical shape and skinny residual limbs.

### AERO liner

The EVA material of the AERO is a packaging material for PE-lite and can be sourced from any major materials and textiles supplier. The AERO liner was prefabricated to specific dimensions: suprapatellar to apex of the distal end of the liner (330 mm) for small, medium and large sizes. Circumference at the apex of the femoral condyles was 330-, 350- and 370-mm. Circumference at the distal 4 cm level: small 240 mm, medium 260 mm and large 280 mm. A full description of the fabrication methods and video instructions is available in [22]. Each participant was evaluated for AERO by measuring their residual limb at 4 cm from the distal end and proximally at the middle of the patella. If the participant presented with a residual limb shape that did not constitute a pre-fabricated fitting, a custom liner was fabricated using methods previously described [22].

### Outcome measurements

#### Pressure uniformity evaluation and socket comfort score (SCS)

**Figure 1.**
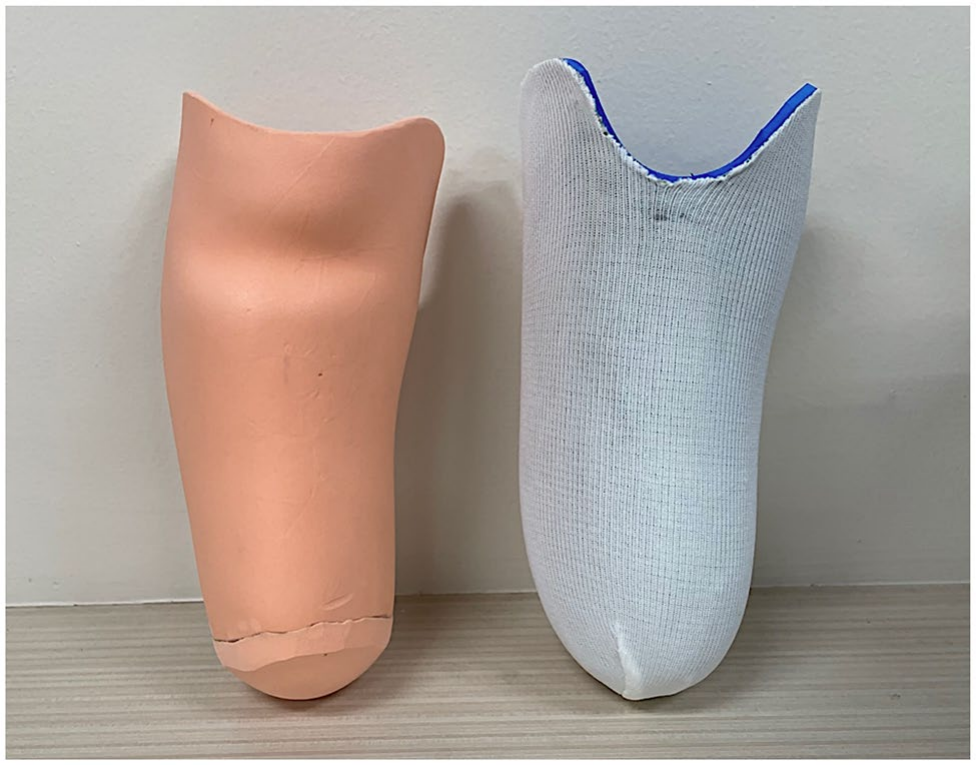
Prosthetic socket liner: (a) PE-lite liner and (b) AERO liner.

**Figure 2.**
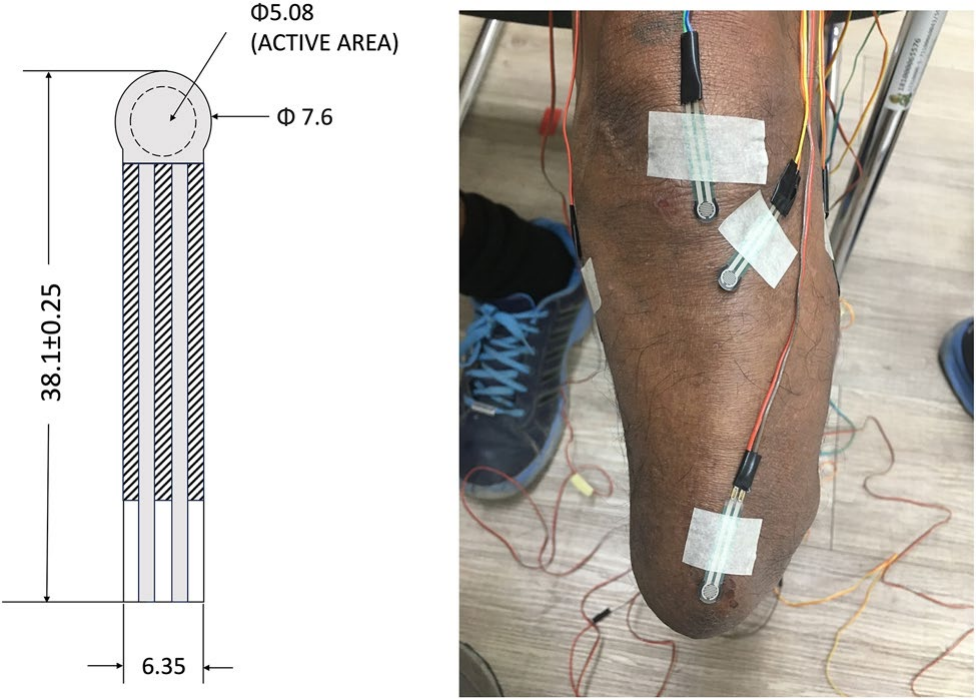
FSR400 Illustration and image of sensor placement on the residual limb.

Flexible force sensors have been widely used to evaluate the pressure on the residual limb and facilitate prosthetic treatment. These sensors help to quantify and compare the pressure patterns between different liner materials, socket designs, or walking conditions. In addition, it provides real-time pressure data to help capture pressure changes during dynamic activities, such as walking [25–29]. The pressure uniformity of the residual limb was measured using a force-sensor resistor (FSR400). Real-time sensor data in kilopascals (kPa) were collected using an Arduino UNO board microcontroller and open-source Arduino IDE 2.0. Data acquisition was performed using a personal computer running Microsoft Windows 10 (Microsoft, Seattle, WA, USA). To ensure accuracy, a precise weight was used for calibration. The voltage data obtained from weights ranging between 20 g and 200 g were subsequently converted to kilopascals. Participants were asked to walk on a single-belt treadmill (Lode, Valiant, Lode B.V., Groningen, The Netherlands) to find their comfortable walking speed. Six force-sensitive resistive (FSR) sensors were directly attached to specific locations on the residual limb: the patella tendon, tibial tuberosity, distal end of the tibia, fibula head, medial tibial flare and posterior region. These areas are known to be sensitive to pressure for transtibial prosthesis users. The patellar tendon, posterior surface and medial tibial flare corresponded to weight-bearing areas created by the patellar tendon-bearing (PTB) socket design, whereas the remaining sensors were placed on bony prominences that primarily serve as pressure-reduction areas (Figure 1). And participants instructed to walk on a single-belt treadmill at their self-selected walking speed (SSWS) for a duration of 2 min. There were two trials for each liner and a 5 min rest for each trial. Forty data steps were used to analyse the pressure distribution [30]. After the 2-min walking test, the subjects were asked to rate the comfort of the socket using the SCS. The socket comfort score is a numerical rating scale used to assess the comfort of a prosthetic. The score ranges from 0 to 10, with 0 being the least comfortable and 10 being the most comfortable [31]. Each liner material was evaluated by the outcome measurements administered by a single researcher (Figure 2).

### Statistics

Statistical analysis was conducted using R statistical software version 4.2.0 (R Project for Statistical Computing) in RStudio statistical software version RStudio 2022.02.2 + 485 ‘Prairie Trillium’. To examine the differences in pressure distribution between the two liner materials, a Wilcoxon signed-rank test was performed. A significant difference was indicated by using a *p* value, smaller than .01 (*p* < .01). The mean peak pressure across all sensors was calculated along with the pooled standard deviation (SD), which was then used to determine the coefficient of variation (CV). A lower CV indicates less divergence between the sensors and greater uniformity in the pressure distribution on the residual limb. In contrast, a higher CV indicated a less uniform limb pressure. To refine this idea, we used the following equation to determine the ‘pressure uniformity’ level based on the CV.

100−(CV×100)%,

This formula converted the CV to a 100% scale for pressure uniformity. 100% indicates perfect pressure uniformity, and 0% indicates no uniformity. The socket comfort score was analysed using the Wilcoxon signed-rank test.

### Material composition test

Understanding the material properties of a liner is essential to ensure safety and transparency. Thus, we performed a Fourier transform infrared (FT-IR) Microscope Perkin Elmer (Spectrum Spotlight 300) to explore PE-lite and local EVA materials. The FTIR is a technique that examines chemical composition of materials by measuring how they absorb infrared light. Different chemical bonds within a material absorb specific frequencies of this light, creating a unique pattern. By analysing these patterns, FTIR can identify the types of chemical bonds present, helping in material analysis, quality control and identifying substances in various industries [32].

## Results

**Figure 3.**
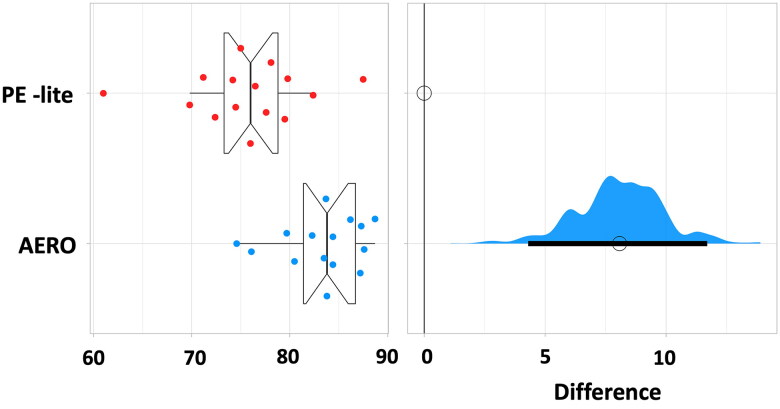
The left graph shows the plot of pressure uniformity between two liner materials of the 15 participants. The right graph displays the effect size relative to PE-lite with a distribution of bootstrap samples for the 95% confidence interval.

**Figure 4.**
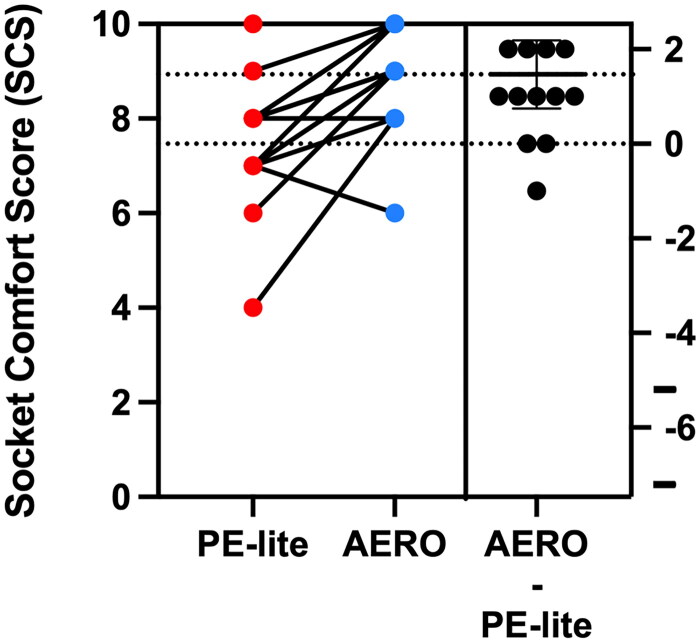
Results of socket comfort score (SCS) for each participant and overall mean difference between devices.

This study included fifteen transtibial prosthetic users, twelve males aged 51.4 ± 9.6 (67.8 kg ± 13.8) and three females aged 62.7 ± 3.2 (59.3 kg ± 16.1). Most of the participants (11) had residual limbs that were well suited for the prefabricated liners; however, some participants (4) needed custom liners (see Table 1). The pressure distribution during walking was analysed, and the CV in PE-lite was 75.7 ± 6.0, and the AERO liner was 83.3 ± 4.1. A Wilcoxon signed-rank test was conducted to examine the differences in pressure distribution between the two liner materials. The analysis revealed a statistically significant difference in the pressure uniformity (*p* = .0007), with a large effect size (*r* = 0.87). The G-power post-hoc analysis indicated a power of 0.86. The mean pressure score for SCS PE-lite was 7.5 with a standard deviation (SD) of 1.3, whereas the mean pressure score for AERO was 8.9 with an SD of 1.1 (see Table 2). The Wilcoxon signed-rank test yielded a *p* value of .003, indicating a significant difference between the two liner materials with an effect size of 0.81 (Figures 3 and 4).

**Table 1.** Participants residual limb shape (*n* = 15).

Subject	Residual limb length	Residual limb shape	AERO liner fabrication
A	Medium	Cylindrical	Pre-fabricated
B	Medium	Conical	Pre-fabricated
C	Medium	Cylindrical	Custom made
D	Short	Cylindrical	Custom made
E	Medium	Conical	Custom made
F	Short	Cylindrical	Pre-fabricated
G	Short	Cylindrical	Pre-fabricated
H	Medium	Cylindrical	Pre-fabricated
I	Medium	Conical	Custom made
J	Short	Conical	Pre-fabricated
K	Medium	Cylindrical	Pre-fabricated

*Notes:* Residual limb length is calculated from the tibia bone length, measured from the mid patella tendon to the malleolus on the sound side and from the mid patella tendon to the distal end of the tibia on the amputated side. The one-third proximal portion of the tibia is categorized as short length, while the middle third of the tibia is categorized as medium length.

**Table 2.** Mean kPa, standard deviation, CV and SCS for PE-lite and AERO liner.

	Pooled mean kPa	Pooled SD	CV	SCS
PE-lite	70.5 (11.9)	16.7 (3.4)	75.7 (6.0)	7.5 (1.3)
AERO	65.8 (10.3)	10.8 (2.0)	83.3 (4.1)	8.9 (1.1)

FTIR tests revealed the compositions of the PE and EVA materials. The main difference between the two liners is the proportion of calcium carbonate (CaCO_3_) in the PE-lite and EVA materials. The EVA material contained a greater amount of CaCO_3_. The chemical composition of each material is presented in Table 3.

**Table 3.** Two different material chemical compositions list.

	PE-lite	Local EVA
Main composition	Ethylene vinyl acetate copolymer	Ethylene vinyl acetate copolymer
	Polyethylene	Polyethylene
		Calcium carbonate (CaCO_3_) Ester compound
Small amount	Calcium carbonate (CaCO_3_)	Magnesium silicate
	Magnesium silicate Ester compound	Primary acid compound
Very small amount	Primary amide compound Silicon dioxide Carboxylic acid compound	Silicon dioxide
Trace amount	Aromatic compound	Aromatic compound
	Calcium stearate	

## Discussion

This study evaluated the pressure distribution of the AERO liner for transtibial prosthesis users and determined its material and chemical properties. The AERO liner provided a more even distribution of pressure across the residual limb and more comfort than PE-lite. This outcome might be a result of the liner material property, which is composed of materials that make for a softer liner than a PE-lite liner, thus enabling roll-on donning that accommodates the residual limb soft tissue. This improved pressure distribution may contribute to reducing the risk of potential issues in the residual limb. It is presumed to be beneficial for individuals who have previously utilized PE-lite and encountered discomfort or skin problems stemming from localized pressure on the residual limb. However, the SCS differences were less than the minimum detectable change (MDC) between PE-lite and the AERO liner, which has an MDC of 2.7 [33]. In our study, the AERO liner was provided to participants with a PTB socket. PTB emphasizes the socket pressure at the proximal region, which can create an unequal pressure distribution on the residual limb. Especially, PE-lite predominantly exerted pressure on the patella tendon with low pressure at the distal part, the AERO liner demonstrated a decrease in patella tendon pressure compared to PE-lite. Additionally, the AERO liner exhibited increased pressure at the distal part. Consequently, the overall pressure, pooled mean and SD decreased when using the AERO liner. These results can be used to illustrate residual limb pressures in either liner type in PTB sockets, with the potential to enhance clinical outcomes for transtibial prosthesis users [34].

An important finding of this study is that the prefabricated AERO was suitable for 11 of the 15 participants. A custom liner was created for the remaining participants. The custom liner does not require casting of the limb; only measurements of the limb are taken, and a pattern is made in approximately 45 min. In essence, the AERO liner could be provided to individuals in need of liner replacement without the need for timely casting or fabrication of a new PE-lite liner. Furthermore, a set of liners can be provided to patients to allow them to take home and use when needed. The EVA material we used in this study originated from Thailand. A single sheet measuring 1*2 m^2^ was employed in developing the prosthesis liner, priced at 8.11 USD per square metre. Step-by-step instructions and video demonstrating the fabrication of the AERO liner can be viewed by referencing our previous technical note [22].

Pressure data were collected for 2 min at a comfortable walking speed on the treadmill. The socket pressure of a patient can be affected by the patient’s walking speed or perspiration from long-distance walking. Residual limb volume fluctuation is one of the challenges faced by prosthesis users. Because PE-lite and AERO data collection in our study was performed on the same day, it is not possible to draw generalizations about longer wear effects [35].

Utilizing a TSB socket with a roll-on liner allows the patient to actively distribute pressure across the limb [36]. This socket may be more suitable for the AERO liner to reduce point pressure on the residual limb. All participants in this study wore PTB sockets with PE-lite, and some participants had observed skin discolouration, particularly in the proximal region, PTB area and fibula head. Numerous discussions have been conducted regarding the efficacy of the PTB prosthetic concept. The PTB bar is not always required if the patient’s residual limb at the distal end can tolerate weight bearing [37]. Furthermore, PTB bars increase the risk of tendon injury [38].

The main constituents of PE-lite include ethylene vinyl acetate copolymer and polyethylene. In contrast, the AERO liner consists of ethylene vinyl acetate copolymer, polyethylene and a higher proportion of calcium carbonate (CaCO_3_). The variance in the main composition of the material may explain the roll-on functionality of the AERO [39]. CaCO_3_ is also a component of talcum powder, which poses a low risk of allergic reactions.

Previously, prosthetists working in RLE were unable to select liner materials to optimize patient prosthesis fitting. In this study, we explored the effects of liners fabricated using affordable materials. The AERO liner has the potential to aid prosthetists and patients by enhancing the efficacy of residual limb pressure distribution. Additionally, AERO may provide more opportunities to offer sockets than benefit from a roll-on liner, such as the TSB socket.

This study has some limitations. The current participants prosthetic components, such as suspension systems and prosthetic feet, may not be conducive for those with high activity levels and faster walking speeds. Consequently, the pressure distribution on the residual limb of a highly active prosthesis user with faster walking speed was unclear. In addition, while all subjects utilized PTB prostheses, the implications of different socket designs, such as TSB or those following the hydrostatic principle, remain uncertain. Furthermore, due to the brief adaptation period, the study lacks insight into the long-term effects on the residual limb and the durability of the liner.

## Conclusion

This study compared the pressure distribution and comfort of the AERO and PE-lite liners and revealed the material composition of the AERO. A comprehensive PTB socket with Pe-lite is widely used in RLE. Gel liners are still costly to employ in RLE. The roll-on donned AERO liner has the potential to enhance the pressure distribution and comfort of these patients. The ability to locally produce an AERO liner makes it an option for the sustainable development of prosthetics.

## Data Availability

The data that support the findings of this study are available from the corresponding author, [K.S].

## References

[1] Yuan B, Hu D, Gu S, et al. The global burden of traumatic amputation in 204 countries and territories. Front Public Health. 2023;11:1258853. doi: 10.3389/fpubh.2023.1258853. 37927851 PMC10622756

[2] Wurdeman SR, Stevens PM, Campbell JH. Mobility analysis of AmpuTees (MAAT 6): mobility, satisfaction, and quality of life among long-term dysvascular/diabetic prosthesis users—results of a cross-sectional analysis. J Prosthet Orthot. 2021;33(3):161–167. doi: 10.1097/JPO.0000000000000304. 34177205 PMC8216599

[3] Klute GK, Glaister BC, Berge JS. Prosthetic liners for lower limb amputees: a review of the literature. Prosthet Orthot Int. 2010;34(2):146–153. doi: 10.3109/03093641003645528. 20384553

[4] Hafner BJ, Cagle JC, Allyn KJ, et al. Elastomeric liners for people with transtibial amputation: survey of prosthetists’ clinical practices. Prosthet Orthot Int. 2017;41(2):149–156. doi: 10.1177/0309364616661256. 27613589 PMC5344787

[5] McDonald CL, Westcott-McCoy S, Weaver MR, et al. Global prevalence of traumatic non-fatal limb amputation. Prosthet Orthot Int. 2021;45(2):105–114. doi: 10.1177/0309364620972258. 33274665

[6] Atlas of amputations and limb deficiencies: surgical, prosthetic, and rehabilitation principles. 4th ed. Rosemont (IL): American Academy of Orthopaedic Surgeons; 2016.

[7] World Health Organization, USAID. 2017 WHO standards for prosthetics and orthotics [Internet]. Geneva: World Health Organization; [cited 2023 Apr 2]. Available from: https://apps.who.int/iris/handle/10665/259209

[8] Wyss D, Lindsay S, Cleghorn WL, et al. Priorities in lower limb prosthetic service delivery based on an international survey of prosthetists in low- and high-income countries. Prosthet Orthot Int. 2015;39(2):102–111. doi: 10.1177/0309364613513824. 24335154

[9] Andrysek J. Lower-limb prosthetic technologies in the developing world: a review of literature from 1994–2010. Prosthet Orthot Int. 2010;34(4):378–398. doi: 10.3109/03093646.2010.520060. 21083505

[10] Al Shuaili N, Aslani N, Duff L, et al. Transtibial prosthetic socket design and suspension mechanism: a literature review. J Prosthet Orthot. 2019;31(4):224–245. doi: 10.1097/JPO.0000000000000258.

[11] Yiğiter K, Sener G, Bayar K. Comparison of the effects of patellar tendon bearing and total surface bearing sockets on prosthetic fitting and rehabilitation. Prosthet Orthot Int. 2002;26(3):206–212. doi: 10.1080/03093640208726649. 12562067

[12] Safari MR, Meier MR. Systematic review of effects of current transtibial prosthetic socket designs—part 1: qualitative outcomes. J Rehabil Res Dev. 2015;52(5):491–508. doi: 10.1682/JRRD.2014.08.0183. 26436666

[13] Safari MR, Meier MR. Systematic review of effects of current transtibial prosthetic socket designs—part 2: quantitative outcomes. J Rehabil Res Dev. 2015;52(5):509–526. doi: 10.1682/JRRD.2014.08.0184. 26436733

[14] Staats T, Lundt J. The UCLA total surface bearing suction below-knee prosthesis. Clin Prosthetics Orthot. 1987;11:118–130.

[15] Baars ECT, Geertzen JHB. Literature review of the possible advantages of silicon liner socket use in trans-tibial prostheses. Prosthet Orthot Int. 2005;29(1):27–37. doi: 10.1080/17461550500069612. 16180375

[16] Cagle JC, Hafner BJ, Sanders JE. Characterization of prosthetic liner products for people with transtibial amputation. J Prosthet Orthot. 2018;30(4):187–199. doi: 10.1097/JPO.0000000000000205. 30906148 PMC6425736

[17] Adigüzel Zengin AC, Örk N, Yiğit Ü, et al. Leather as a potential liner for the prosthetic leg users. LFJ. 2018;18(4):321–326. doi: 10.24264/lfj.18.4.7.

[18] Sanders JE, Nicholson BS, Zachariah SG, et al. Testing of elastomeric liners used in limb prosthetics: classification of 15 products by mechanical performance. J Rehabil Res Dev. 2004;41(2):175–186. doi: 10.1682/jrrd.2004.02.0175. 15558371

[19] Yang X, Zhao R, Solav D, et al. Material, design, and fabrication of custom prosthetic liners for lower-extremity amputees: a review. Med Nov Technol Dev. 2023;17:100197. doi: 10.1016/j.medntd.2022.100197.

[20] Boutwell E, Stine R, Hansen A, et al. Effect of prosthetic gel liner thickness on gait biomechanics and pressure distribution within the transtibial socket. J Rehabil Res Dev. 2012;49(2):227–240. doi: 10.1682/jrrd.2010.06.0121. 22773525

[21] Bui KM, Raugi GJ, Nguyen VQ, et al. Skin problems in individuals with lower-limb loss: literature review and proposed classification system. J Rehabil Res Dev. 2009;46(9):1085–1090. doi: 10.1682/jrrd.2009.04.0052. 20437314

[22] Sasaki K, Guerra G, Rattanakoch J, et al. Sustainable development: a below-knee prostheses liner for resource limited environments. J Med Dev. 2020;14(1):014501. doi: 10.1115/1.4045835.

[23] Miyata Y, Sasaki K, Guerra G, et al. Sustainable, affordable and functional: reimagining prosthetic liners in resource limited environments. Disabil Rehabil. 2022;44(12):2941–2947. doi: 10.1080/09638288.2020.1844316. 33167733

[24] Gailey RS, Roach KE, Applegate EB, et al. The amputee mobility predictor: an instrument to assess determinants of the lower-limb amputee’s ability to ambulate. Arch Phys Med Rehabil. 2002;83(5):613–627. doi: 10.1053/apmr.2002.32309. 11994800

[25] Parmar S, Khodasevych I, Troynikov O. Evaluation of flexible force sensors for pressure monitoring in treatment of chronic venous disorders. Sensors. 2017;17(8):1923. doi: 10.3390/s17081923. 28825672 PMC5580323

[26] Swanson EC, Weathersby EJ, Cagle JC, et al. Evaluation of force sensing resistors for the measurement of interface pressures in lower limb prosthetics. J Biomech Eng. 2019;141(10):1010091–10100913. doi: 10.1115/1.4043561. 31017621 PMC6808001

[27] Al-Fakih E, Abu Osman N, Mahmad Adikan F. Techniques for interface stress measurements within prosthetic sockets of transtibial amputees: a review of the past 50 years of research. Sensors. 2016;16(7):1119. doi: 10.3390/s16071119. 27447646 PMC4970162

[28] Hopkins MO, Turner S, Vaidyanathan R, et al. Mapping lower-limb prosthesis load distributions using a low-cost pressure measurement system. Front Med Technol. 2022;4:908002. doi: 10.3389/fmedt.2022.908002. 35782578 PMC9247243

[29] Rajtukova V, Hudak R, Zivcak J, et al. Pressure distribution in transtibial prostheses socket and the stump interface. Proc Eng. 2014;96:374–381. doi: 10.1016/j.proeng.2014.12.106.

[30] Sasaki K, Guerra G, Lei Phyu W, et al. Assessment of socket pressure during walking in rapid fit prosthetic sockets. Sensors. 2022;22(14):5224. doi: 10.3390/s22145224. 35890905 PMC9319515

[31] Hanspal R, Fisher K, Nieveen R. Prosthetic socket fit comfort score. Disabil Rehabil. 2003;25(22):1278–1280. doi: 10.1080/09638280310001603983. 14617445

[32] Bhokare SS, Biradar VR, Chakole RD, et al. Applications of FTIR spectroscopy. Review. 2022;7:213–219.

[33] Hafner BJ, Morgan SJ, Askew RL, et al. Psychometric evaluation of self-report outcome measures for prosthetic applications. J Rehabil Res Dev. 2016;53(6):797–812., CPOdoi: 10.1682/JRRD.2015.12.0228. 28273329 PMC5345485

[34] Sewell P, Noroozi S, Vinney J, et al. Static and dynamic pressure prediction for prosthetic socket fitting assessment utilising an inverse problem approach. Artif Intell Med. 2012;54(1):29–41. doi: 10.1016/j.artmed.2011.09.005. 21963113

[35] Sanders JE, Cagle JC, Allyn KJ, et al. How do walking, standing, and resting influence transtibial amputee residual limb fluid volume? J Rehabil Res Dev. 2014;51(2):201–212., BSEdoi: 10.1682/JRRD.2013.04.0085. 24933719 PMC4435803

[36] Dumbleton T, Buis AWP, McFadyen A, et al. Dynamic interface pressure distributions of two transtibial prosthetic socket concepts. JRRD. 2009;46(3):405. doi: 10.1682/JRRD.2008.01.0015. 19675992

[37] Abu Osman NA, Spence WD, Solomonidis SE, et al. The patellar tendon bar! Is it a necessary feature? Med Eng Phys. 2010;32(7):760–765. doi: 10.1016/j.medengphy.2010.04.020. 20678997

[38] Ho K-Y, Harty M, Kellogg J, et al. Patellar tendon morphology in trans-tibial amputees utilizing a prosthesis with a patellar-tendon-bearing feature. Sci Rep. 2019;9(1):16392. doi: 10.1038/s41598-019-52747-9. 31704989 PMC6841932

[39] Öksüz M, Yıldırım H. Effect of calcium carbonate on the mechanical and thermal properties of isotactic polypropylene/ethylene vinyl acetate blends. J Appl Polym Sci. 2005;96(4):1126–1137. doi: 10.1002/app.21555.

